# Does mixing of beech (*Fagus sylvatica*) and spruce (*Picea abies*) litter hasten decomposition?

**DOI:** 10.1007/s11104-013-2001-9

**Published:** 2013-12-20

**Authors:** Torsten W. Berger, Pétra Berger

**Affiliations:** Department of Forest- and Soil Sciences, Institute of Forest Ecology, University of Natural Resources and Live Sciences (BOKU), Peter Jordan-Straße 82, 1190 Vienna, Austria

**Keywords:** Decomposition, *Fagus sylvatica*, Litter quality, Litterbag, Mixing effects, *Picea abies*

## Abstract

**Background and aims:**

It is of practical relevance to know how much beech must be admixed to pure spruce stands in order to increase litter decomposition and associated nutrient cycling, since the formation of thick organic layers is commonly ascribed to the recalcitrance of spruce needles. We addressed the impact of tree species mixture within forest stands and within litter on mass loss and nutritional release from litter.

**Methods:**

Litter decomposition was measured in three adjacent stands of pure spruce (*Picea abies*), mixed beech-spruce and pure beech (*Fagus sylvatica*) on a nutrient-rich site and a nutrient-poor site over a 2-year period using litterbags which were filled with five different mixtures of beech and spruce litter.

**Results:**

Mass loss of beech litter was not higher than mass loss of spruce litter. Decay was primarily affected by tree species composition of the incubation stand and was faster in (mixed) beech forests stands than in spruce forests, while the influence of litter species and their mixtures on decay rates was small. Net transfers of nutrients between the two litter species (direct effects) in the mixed bags were minimal, since initial beech and spruce litter did not have different litter quality. However, in a few cases indirect effects (e.g., changing decomposer abundance and activity) caused non-additive patterns for the totals within the mixed bags, hastening decomposition within the first year.

**Conclusions:**

Greater accumulation of litter in spruce compared to beech stands is not a consequence of the inherent recalcitrance of needles. Adverse environmental conditions in spruce stands retard decomposition. Indirect effects on decomposition caused by stand mixture are not mimicked by litter mixtures within mesh bags.

## Introduction

Replacement of beech by spruce is associated with changes in soil acidity, soil structure and humus form, which are commonly ascribed to the recalcitrance (e.g., high C/N ratios and lignin concentrations) of spruce (e.g., Ellenberg et al. 1986). The formation of thick organic layers in monocultures of spruce is associated with reduced tree growth and therefore “hampers forest productivity” (Kazda and Pichler 1998). Hence, knowing how much beech must be admixed to pure spruce stands in order to increase litter decomposition, is of practical relevance for forest management strategies, since conversion of secondary pure spruce stands to mixed species stands is a current issue in Europe (Spiecker et al. 2004).

Decomposition processes are important for cycling of nutrients in forest ecosystems and are influenced by macro- and micro-climate, litter quality, activity of decomposing organisms and soil nutrient status (Vesterdal 1999). Mixing litter from species with differing resource quality and leaf structure changes the chemical environment and physically alters the total litter surface where decomposition is occurring (Hector et al. 2000). These alterations can also affect decomposer abundance and activity (Scheu et al. 2003). Thus, chemical and physical changes in leaf mixtures can influence decomposition rates both directly (physically) and indirectly (through the decomposer community and its activities). Gartner and Cardon (2004) found 30 papers that focus directly on decomposition of mixtures of litters, assessing whether decay rates in species mixtures can be predicted from known decay rates of the component litters (additive effects) decaying alone, but, i.e., not a single paper in this review explored decomposition of mixed beech-spruce litter, simultaneously examining the decay of the component single species. The term “decomposition”, used in this study, comprises both mass loss (decay rate) and nutrient release (including nutrient transfers among leaves of different species), which are not necessarily linked with each other. The review by Gartner and Cardon (2004) revealed that nutrient transfer among leaves of different species is striking, with 76 % of the mixtures showing non-additive dynamics of nutrient concentrations. In accordance with the comprehensive work of Wardle et al. (1997) these non-additive effects of decomposing mixed litter are difficult to generalize. Whether nutrient transfers within the decomposing litters are mediated by physical (e.g., leaching) or biological (e.g., fungi) means, nutrients released from rapidly decaying, higher-quality litter can stimulate decay in adjacent, more recalcitrant litters (McTiernan et al. 1997; Sariyildiz et al. 2005) or conversely, leaf litter decay can be slowed by release of inhibitory compounds such as phenolics including tannins (Fyles and Fyles 1993; Prescott et al. 2000). There are also recent indications that decay rates of litter mixtures may display additive characteristics (Vivanco and Austin 2008; Hoorens et al. 2010; Jacob et al. 2010).

In accordance with Prescott et al. (2004) the long-held belief that slow decomposition of spruce needle litter is responsible for the formation of thick organic layers and reduced productivity in spruce stands relative to beech forests needs critical testing: “Although we do not actively manage litter decomposition, several assumptions about decomposition are implicit in our expectations. For example, we expect that adding or increasing the broadleaf component will improve the site by increasing nutrient cycling and availability, partly through its higher quality litter and faster decay”. Hence, in a recent publication (Berger and Berger 2012), we focused on purported “safe” generalizations about beech litter and its decomposition in single and mixed (50:50 %) spruce-beech litter combinations. We measured litter decomposition in three adjacent stands of pure spruce (*Picea abies*), mixed beech-spruce and pure beech (*Fagus sylvatica*) on three nutrient-rich sites (bedrock: Flysch) and three nutrient-poor sites (bedrock: Molasse; yielding a total of 18 stands) over a 3-year period using the litterbag method and addressed the impact of tree species composition within forest stands and litter. Since this research is a complementary study to the previous one, citing short summaries of the answers to the questions asked by Berger and Berger (2012) bears repeating at this point: The expectation, that broadleaf components decay faster, was not fulfilled (question 1: Does beech litter decompose faster than spruce litter?). Decay (mass loss) and release of compounds building up the organic litter layer (C_org_, N_tot_, P, S and lignin) and associated K were primarily affected by tree species composition of the incubation stand and were faster in (mixed) beech forest stands than in spruce forests (question 2: Does litter decompose faster in beech or mixed beech-spruce forests than in spruce forests?). Mass loss did not differ between single and mixed spruce litter or between single and mixed beech litter but mixing beech and spruce litter tended to increase decay of spruce needles after the first year (question 3: Does mixing of beech and spruce litter hasten decomposition of spruce litter?). Mass loss was driving the release of the main components of the organic substance (C_org_, lignin, N_tot_, P and S) and associated K but nutrient release of the base cations (except K) and Mn and Fe was not related to mass loss (question 4: Does mass loss correlate with nutrient release?). The initial element content in the litter explained most of the variation in the release of the same element for P, Mg, K, Na and lignin; the (non-chemical) soil environment (e.g., micro-climate, physical conditions, activity of decomposing organisms) primarily controlled the decomposition rate *k* (mass loss) and C_org_ release and to a lesser extent release of N_tot_, S and lignin; soil nutrition parameters helped to explain part of the remaining variance in release of S, Mg, K and lignin (question 5: Which parameters represent the best suite of characteristics that actually control decay rates and nutrient release?).

Within the overall approach (see above; Berger and Berger 2012) this study was designed to pick up Question 3 in more details: Does mixing of beech and spruce litter hasten decomposition? Again, we measured litter decomposition in three adjacent stands of pure spruce, mixed beech-spruce and pure beech but only at the most intensively studied experimental site of each substrate for soil formation: Kreisbach (nutrient-rich site on Flysch) and Frauschereck (nutrient-poor sites on Molasse; yielding a total of six stands) over a 2-year period. However, this time, litter bags were filled with five instead of three different mixtures of spruce and beech litter enabling supplementary methods to test the hypothesis that decomposition and nutrient release of foliage litter of beech and spruce indicates non-additive effects of litter mixtures. Since separate sets of litterbags were exposed over a deviating time period this study can be used to verify the conclusion of Berger and Berger (2012) that “greater accumulation of litter in spruce compared to beech stands (see Question 2 above) is not a consequence of the inherent recalcitrance of needles (see Question 1 above)”.

## Materials and methods

### Study sites

Six sites were selected on the two different bedrocks Flysch and Molasse (3 comparable sites on each substrate). Beech and spruce were similarly mixed, before one stand at each site was converted into the current pure spruce stand. Mono specific beech stands (5–7 canopy dominant trees) were selected within the mixed species stands. Nutrient fluxes had been monitored for the same 18 stands and detailed site information is given by Berger et al. (2009a) for each of the 18 stands. The stand characteristics of the sites Kreisbach (Flysch) and Frauschereck (Molasse) are listed in Table 1, since this specific study on litter decomposition was performed only on these sites, which had been extensively studied before (Schmid and Kazda 2001, 2002; Berger et al. 2004b, 2006, 2009b, 2010). Standing timber volume and dominant tree heights are higher at Kreisbach, despite a somewhat younger stand age of beech. The stands are located on N- (Kreisbach) to W- (Frauschereck) facing slopes. Precipitation declines from the western (Molasse) to the eastern (Flysch) parts of Austria.

**Table 1:** Forest stand characteristics of adjacent pure and mixed species stands at the experimental sites Kreisbach and Frauschereck according to a 1997 survey, modified from Berger et al. 2009a. Since mono specific beech stands (5–7 canopy dominant trees) were selected within the mixed species stands at Frauschereck, ha-related stand characteristics are the same for the mixed and the pure beech stand. At Kreisbach, the pure beech stand was large enough for a separate survey

Site	Age	Stems	Timber volume	Basal area	Dominant tree height	Elevation	Slope	Aspect (from N to E)	Precipitation (1971–2000)	Coordinates (WGS84)	
	Years	N ha^−1^	m^3^ ha^−1^	m^2^ ha^−1^	m	m a.s.l.	Degrees	Degrees	mm	N	E
Kreisbach											
Spruce	53	1012	567	57	27.0	480	11	0.0	850	48°05′50″	15°39′46″
Mixed	65	976	487	44	27.5	480	11	0.0	850	48°05′50″	15°39′49″
Beech	65	960	588	47	28.0	480	11	0.0	850	48°05′50″	15°39′54″
Frauschereck											
Spruce	58	1264	432	51	22.0	710	8	292.5	1180	48°05′27″	13°18′36″
Mixed	89	414	384	42	29.5	700	7	292.5	1180	48°05′33″	13°18′39″
Beech	89	414	384	42	28.0	690	7	315.0	1180	48°05′35″	13°18′36″

Detailed site descriptions are given by Berger et al. (2009a). The substrate for soil formation at Kreisbach is Flysch. The Flysch zone is a narrow strip in the foothills of the Northern Limestone Alps from west to east throughout the country. Flysch consists mainly of old tertiary and mesozoic sandstones and clayey marls. Nutrient release from this bedrock is high and consequently the prevalent humus forms are mull (beech and mixed stands; less than 1 cm thickness) to intermediate types between mull and moder (pure spruce stand), indicating quick turnover of the forest floor (usually less than 2 cm thick). Soil parameters (Table 2) indicate nutrient-rich soils. Soils were classified as pseudogley (Scheffer and Schachtschabel 1998; FAO classification: stagnic cambisol), since horizons with a high fraction of fine material (loam to clay) cause temporary waterlogging (stagnation zone at approximately 40–50 cm soil depth). There are hardly any shrubs and the total cover of the herb layer is between 5 % (spruce) and 20 % (beech). The natural forest vegetation of the mixed stands on Flysch is *Asperulo odoratae-Fagetum* (Mucina et al. 1993).

**Table 2:** Mean soil properties of the forest floor, 0–10 cm mineral soil and top soil (forest floor + 0–10 cm mineral soil) under the pure and mixed incubation stands of spruce and beech at the sites Kreisbach and Frauschereck: total stores of C_org_ (kg m^−2^ per horizon) and C_mic_, N_tot_, P and S (g m^−2^ per horizon); stores of Ca, Mg, K, Na, Al, Fe and Mn, given as total content in the forest floor and exchangeable content in the mineral soil (g m^−2^ per horizon); cation exchange capacity (CEC), sum of acid cations (Al, Fe, Mn, H^+^) in mol_c_ m^−2^ per horizon; base saturation (%); C_org_/N_tot_ and C_org_/P ratio; C_mic_ in percent of C_org_

Incubation stand	C_org_	C_mic_	N_tot_	P	S	Ca	Mg	K	Na	Al	Fe	Mn	CEC	Acid cat.	Base sat.	C_org_/N_tot_ ratio	C_org_/P ratio	C_mic_/C_org_ (%)
Kreisbach																		
Forest floor																		
Spruce	0.91	12	34	2.3	4.0	22.4	20.9	24.9	1.3	95.4	82.9	11.0	19.0	15.5	18.6	26.7	390.3	1.3
Mixed	0.36	4	11	0.5	1.1	11.1	3.7	5.2	0.3	15.9	12.1	2.4	3.5	2.5	28.5	33.8	779.8	1.0
Beech	0.41	9	14	0.8	1.4	20.2	4.7	6.5	0.4	17.5	12.4	2.3	4.3	2.7	36.9	30.2	497.2	2.1
0–10 cm																		
Spruce	3.04	42	237	29.3	32.9	77.7	6.6	21.4	6.1	48.5	0.0	5.7	10.8	5.6	48.2	12.9	103.9	1.4
Mixed	2.42	48	105	28.2	27.2	64.8	6.8	21.3	8.0	43.6	0.0	2.9	9.7	5.0	48.6	23.0	85.7	2.0
Beech	2.07	53	62	32.0	25.3	82.7	10.0	28.8	7.1	25.0	0.0	1.1	8.8	2.8	68.0	33.7	64.9	2.5
Top soil																		
Spruce	3.95	53	270	31.6	36.9	100.0	27.5	46.3	7.5	143.9	82.9	16.7	29.8	21.1	29.4	14.6	124.9	1.3
Mixed	2.78	52	116	28.7	28.3	75.9	10.5	26.4	8.2	59.5	12.1	5.3	13.2	7.5	43.2	24.0	96.9	1.9
Beech	2.49	61	75	32.8	26.7	103.0	14.7	35.2	7.5	42.5	12.4	3.4	13.1	5.5	57.8	33.0	75.9	2.5
Frauschereck																		
Forest floor																		
Spruce	5.37	46	207	11.1	28.5	29.0	34.1	36.1	5.4	295.7	292.2	4.7	54.2	48.8	10.0	26.0	482.4	0.9
Mixed	4.66	33	178	7.9	23.8	25.5	21.6	29.5	4.7	181.6	143.7	3.5	32.1	28.1	12.5	26.2	592.0	0.7
Beech	3.14	26	142	6.8	16.4	46.5	28.6	36.7	5.6	221.5	178.4	4.0	40.2	34.4	14.6	22.1	460.9	0.8
0–10 cm																		
Spruce	3.46	37	140	26.3	23.6	0.7	0.4	3.3	4.4	47.5	0.1	0.1	5.7	5.3	6.0	24.7	131.5	1.1
Mixed	3.72	44	189	25.1	30.2	0.4	0.7	6.3	3.7	55.9	0.2	0.1	6.7	6.2	6.1	19.7	147.9	1.2
Beech	3.45	35	147	19.1	29.1	4.4	0.9	5.4	4.6	39.4	0.1	0.1	5.0	4.4	12.5	23.5	180.7	1.0
Top soil																		
Spruce	8.83	83	347	37.5	52.1	29.7	34.5	39.4	9.8	343.2	292.3	4.7	59.8	54.1	9.6	25.4	235.8	0.9
Mixed	8.38	77	367	33.0	54.0	25.9	22.3	35.8	8.4	237.4	143.9	3.6	38.7	34.3	11.4	22.9	253.9	0.9
Beech	6.59	61	289	25.9	45.5	50.9	29.5	42.1	10.2	260.9	178.5	4.0	45.3	38.8	14.3	22.8	254.4	0.9
Factor site (Top soil)																		
Kreisbach	3.07**	55^(^*^)^	154*	31.0 ns	30.6**	93.0**	17.6 ns	36.0 ns	7.7*	82.0*	35.8*	8.5 ns	18.7*	11.4*	43.5*	23.9 ns	99.2**	1.9*
Frauschereck	7.94	74	334	32.1	50.5	35.5	28.8	39.1	9.5	280.5	204.9	4.1	47.9	42.4	11.8	23.7	248.0	0.9

A one-way ANOVA (factor site; *N* = 2 sites × 3 incubation stands = 6) was done to test mean differences between the sites Kreisbach and Frauschereck for the top soil; level of significance is shown as: ns: not significant, *p* > 0.10; ^(^*^)^: *p* < 0.10; *: *p* < 0.05; **: *p* < 0.01; ***: *p* < 0.001

Parent material for soil formation at Frauschereck is Molasse, tertiary sediments (so-called “Hausruck-Kobernausserwald” gravel), which consist mainly of quartz and other siliceous material (granite, gneiss, hornblende schist, pseudotachylite and colored sandstone). Because of this acidic bedrock with low rates of nutrient release, the dominant soil types are mainly semi-podzols (Scheffer and Schachtschabel 1998; intermediate soil type between cambisol and podzol; FAO classification: dystric cambisol). Humus form is acidic moder and the thickness of the forest floor layers varies between 8 and 10 cm, indicating slow turnover and accumulation of nutrients. In general, soils on Molasse contain more organic carbon and are more acidic, more sandy and less supplied with nutrients than soils on Flysch (Table 2). There are no shrubs and the total cover of the herb layer is 10 % (spruce) to 15 % (beech). The natural forest vegetation of the mixed stands is *Luzulo nemorosae-Fagetum* (Mucina et al. 1993).

### Soils

Mean soil parameters within the overall approach (18 stands) are given by Berger and Berger (2012) and the specific data for the sites Kreisbach and Frauschereck are listed in Table 2. Forest floor (O-horizon: O_i_ + O_e_ + O_a_) and mineral soil (0–10 cm) were taken with a core sampler of 70 mm diameter in summer 2006. There were three distributed replicate samples at each stand, which were pooled before analysis. Samples of forest floor and of mineral soil (fine soil, separated by sieving <2 mm) were analyzed for total content of C (LECO SC 444, USA), N (Kjeldahl method according to ÖNORM L1082; 2300 Kjeltec Analyzer Unit, Tecator, Sweden), P and S (both after digestion with HNO_3_/HClO_4_ according to ÖNORM L1085; ICPS, inductive coupled plasma spectrometry, Optima 3000 XL, Perkin Elmer, USA). Organic carbon (C_org_) was calculated as total carbon minus C_CaCO3_ (Scheibler method: reaction of carbonates with HCl and volumetric determination of emerging CO_2_ according to ÖNORM L1084). Calcium, Mg, K, Na, Al, Fe and Mn were measured as total contents after digestion with HNO_3_/HClO_4_ in the forest floor and as exchangeable cations (0.1 M BaCl_2_ extract) in the mineral soil by ICPS. Soil acidity was measured as pH with a glass Ag/AgCl combination electrode with KCl reference electrode (10 g soil were mixed with 25 ml of 0.01 M CaCl_2_ or deionized H_2_O, stirred, and the pH was measured the next morning 30 min after stirring again). Elemental stocks were then calculated as the product of dry (105 °C) fine-soil masses (related to area and soil depth) and corresponding element contents. Microbial C (C_mic_) was calculated as the difference in organic C between fumigated and non-fumigated (control) samples according to Schinner et al. (1996). Two replicates of each sample, 2.5 g fresh forest floor or 5 g fresh mineral soil, were fumigated for 24 h with ethanol-free chloroform at 25 °C. Subsequently the chloroform was removed by evacuation. Fumigated samples and controls were extracted with 25 ml 0.5 M K_2_SO_4_ and filtered; extracts were kept frozen until analysis. Total dissolved organic carbon was analyzed in the extracts with a Shimadzu TOC-5050 Total Carbon Analyzer, Japan. Non-extracted amounts of microbial C were compensated for by a correction factor of k_EC_ = 0.35.

### Litterbag experiment

Litter of beech and spruce was collected by spreading nets from mid-September to late October 2005 under the pure stands of beech and spruce. Collected foliage litter was dried at 50 °C for 48 h, however, all data in this paper are related to 105 °C dry weight, estimated from subsamples not used for the decomposition study.

Strips of polyethlylene nets (1-mm mesh) were folded to obtain double-layered litterbags (10 × 10 cm size), which were closed on the two open sides with high carbon steel paper-clips. The litterbags were filled with five different mixtures, yielding eight components to be analyzed: mixture 1: single spruce, component SP; mixture 2: spruce-beech ratios of 0.75:0.25, components mSP(0.25BE) [= spruce needles of mixture 2] and mBE(0.75SP) [= beech leaves of mixture 2]; mixture 3: spruce-beech ratios of 1:1, components mSP(0.50BE) and mBE(0.50SP); mixture 4: spruce-beech ratios of 0.25:0.75, components mSP(0.75BE) and mBE(0.25SP); mixture 5: single beech, component BE. The single spruce bags were filled with 2 g, the single beech bags with 3 g and all mixed bags with a total of 4 g of dried (50 °C) litter. On an area basis these litter amounts (195–390 g m^−2^; related to 105 °C) represent the lower range of annual litter input (370–560 g m^−2^ year^−1^ at Kreisbach; 310–370 g m^−2^ year^−1^ at Frauschereck; Berger et al. 2009b).

Beginning of December 2005 the litterbags were placed on the forest floor (after stripping off part of the non decayed leaves and needles of the O_i_-layer and covering the bags thereafter again) in a randomized block design with four 0.4 × 0.7 m blocks per stand. Each of the blocks contained two sets of the five litter mixtures for sampling at two different dates. The bags of each set were connected with each other by two strings along the left and right sites, tied to one wooden stick above and below each block. In addition, each individual bag was fastened to the forest floor by one 10 cm long pin of high carbon steel on the left and right side, outside the clipped seam. A total of 240 litterbags were used for the entire study (2 sites × 3 incubation stands × 5 litter mixtures × 4 replications per stand × 2 sampling dates = 240). Litter bags were collected twice after 1 and 2 years in November. Each set was returned horizontally in flat, piled-up boxes to avoid mass loss via transport. After drying at 40 °C the bags were opened, non-foliage litter material was sorted out and the mixed bags were separated into its components by hand. Thereafter, the components of each individual bag were dried at 105 °C for 48 h, weighed and the 4 block replicates were subsequently pooled to give one sample and were ground for chemical analysis.

Contents of initial litter and after 1 and 2 years in the pooled litter samples were analyzed for C_org_, N_tot_, P, S, Ca, Mg, K, Na, Al, Fe and Mn as described for the soil (forest floor) samples above. Total lignin content (acid-insoluble lignin plus acid-soluble lignin) was measured by Fourier transform near infrared (FT-NIR) spectrometry (Bruker FT-IR spectrometer, EQUINOX 55, Germany, equipped with NIR fibre optic (measuring the diffuse reflected light)) and a germanium-diode detector, limited by a cut-off wavenumber of 5100 cm^−1^ (details of the method are given in Schwanninger et al. 2009). This indirect method proved to be a powerful tool for rapid estimation of the lignin content in agreement with direct classical wet-lab chemistry data (Schwanninger and Hinterstoisser 2002). Microbial C (C_mic_) was measured only in the initial samples as done for the fresh forest floor samples.

### Data evaluation and statistics

Mass loss was calculated as the difference between the initial dry mass and the actual dry mass at each sampling date. Nutrient release was estimated as initial content minus content at each sampling date and expressed either in % of the initial content or in mg g^−1^ incubated litter.

One-way ANOVAs were performed to test whether significant differences of soil properties (site) and initial litter chemistry (litter mixture, site) were caused by the corresponding grouping variables (given in parentheses). The largest data set was used for the performance of a three-way (2 × 3 × 8) ANOVA to test effects of site (nutrient-rich soils at Kreisbach versus nutrient-poor soils at Frauschereck), incubation stand (spruce, mixed, beech) and litter mixture (single spruce, SP; mixed spruce with 25 % [mSP(0.25BE)], 50 % [mSP(0.50BE)] and 75 % [mSP(0.75BE)] admixture of beech; mixed beech with 25 % [mBE(0.25SP)], 50 % [mBE(0.50SP)] and 75 % [mBE(0.75SP)] admixture of spruce; single beech, BE) on the remaining mass (% of initial value) for each sampling date (*N* = 2 sites × 3 incubation stands × 8 litter mixture components × 4 replications per stand = 192). In case of significant interactions between the grouping factors these factors can not be tested individually but affect the dependant factor jointly. Differences between the group means of each litter mixture component were compared by Duncan multiple range tests. Because the 4 replications per stand were pooled before chemical analysis the same three-way ANOVA could not be performed on the remaining element contents (% of initial value), nutrient release (mg g^−1^) and selected compound ratios of litter enclosed in mesh bags due to a shortage of degrees of freedom. Hence, our best estimate was achieved by splitting up the three-way ANOVA into a two-way ANOVA (factors site and incubation stand) and a one-way ANOVA (factor litter mixture) and comparison of means by a Duncan multiple range test.

Predicted remaining masses and nutrient contents were calculated for each of the two mixed species within the mixed bags by linear interpolation from the single species mixtures of spruce and beech according to the initial ratio of mass and nutrient content, respectively. Significant differences (paired *t*-test) between predicted and measured total (mixed spruce + mixed beech within a bag) values in the mixed litter bags of all 3 combinations (25, 50 and 75 % admixture of beech) were calculated at the stand level (grouped by sampling date, site and incubation stand; *N* = 3 mixed bags; indicated as positive or negative in complex figures) and on the site level (*N* = 3 incubation stands × 3 mixed bags = 9). For the latter case, differences between predicted and measured values for specific mixtures were compared by a Duncan multiple range test. According to this method, mass loss and nutrient release, respectively, can be predicted from the component litters decomposing alone (additive effects) for all cases the paired *t*-test does not indicate significant differences. All statistics were performed with the package PASW Statistics 17 (Release 17.0.2, 11 March 2009).

## Results

### Soils

Soil properties of the top soil (forest floor + 0–10 cm mineral soil) indicated significant differences between the soils at Kreisbach and Frauschereck for all listed parameters (Table 2) except stores of P, Mg, K and Mn and the C_org_/N_tot_ ratio. Since a slower turnover causes higher accumulation of the forest floor at Frauschereck, nutrient stores were significantly enriched for all elements except Mn (statistics are not shown in Table 2). The forest sites at Kreisbach had higher base saturation, higher C_mic_/C_org_ ratios but lower C_org_/P ratios within the top soil. Comparing individual base cation storages within the 0–10 cm mineral soil (significantly higher for Ca, Mg, K and Na at Kreisbach; not shown in Table 2), justifies calling soils at Kreisbach nutrient-rich and soils at Frauschereck nutrient-poor.

As documented elsewhere (Berger et al. 2002, 2004a), acidifying effects of spruce were more pronounced on soils formed over Flysch than on Molasse. No statistics could be performed for the single sites, but there is a clear trend that even at both sites base saturation is increasing and the sum of aicid cations is decreasing from spruce to mixed to beech stands. Soil pHs (CaCl_2_) at 0–10 cm from spruce (3.5; 3.3) to mixed (3.7; 3.1) to beech (4.0; 3.1) illustrate soil acidifying effects of spruce at Kreisbach (first value) but not at Frauschereck (second value within parentheses; not shown in Table 2).

### Initial litter quality

Initial element contents and ratios of lignin/N_tot_, C_org_/N_tot_, C_org_/P and C_mic_/C_org_ of spruce (SP) and beech (BE) litter, collected at adjacent spruce and beech stands at Kreisbach and Frauschereck in fall 2005, are given in Table 3. There is a clear trend that the base cation (Ca, Mg and K) and C_mic_ contents and the C_mic_/C_org_ ratio are higher in beech than in spruce. In all other cases differences between beech and spruce were negligible or indicated even lower quality of beech litter (i.e.: higher lignin and higher lignin/N_tot_ ratios for beech than for spruce; Table 3). These data are in accordance with mean chemistry of initial litter, collected in fall 2004 within the overall approach at 3 sites on Flysch and Molasse, respectively, by Berger and Berger (2012). Surprisingly, the different nutritional status of soils at Kreisbach and at Frauschereck (Table 2) was not reflected in initial litter chemistry (factor site; Table 3).

**Table 3:** Initial nutrient contents (mg g^−1^), ratios of lignin/N_tot_, C_org_/N_tot_ and C_org_/P and C_mic_ in percent of C_org_ of spruce (SP) and beech (BE) litter, collected at adjacent spruce and beech stands at the sites Kreisbach and Frauschereck in fall 2005

Litter mixture	C_org_	C_mic_	Lignin	N_tot_	P	S	Ca	Mg	K	Na	Al	Fe	Mn	Lignin/N_tot_ ratio	C_org_/N_tot_ ratio	C_org_/P ratio	C_mic_/C_org_ (%)
Kreisbach																	
SP	494.8	0.7	273.6	8.5	0.5	0.8	12.2	0.7	3.4	0.1	0.2	0.2	2.5	32.3	58.4	923.2	0.2
BE	495.0	8.2	392.8	9.7	0.6	1.2	14.7	1.7	6.1	0.1	0.4	0.3	1.1	40.3	50.8	838.6	1.7
Frauschereck																	
SP	511.6	0.4	285.6	9.5	0.7	0.8	4.6	0.5	2.1	0.2	0.2	0.1	1.0	30.0	53.7	779.2	0.1
BE	504.4	14.5	442.6	7.6	0.7	1.0	11.6	1.2	3.1	0.1	0.1	0.1	1.1	58.4	66.6	718.4	2.9
Factor litter mixture																	
SP (All)	503.2	0.6^(^*^)^	279.6*	9.0	0.6	0.8^(^*^)^	8.4	0.6^(^*^)^	2.7	0.2	0.2	0.1	1.7	31.2	56.1	851.2	0.1^(^*^)^
BE (All)	499.7	11.4	417.7	8.7	0.6	1.1	13.2	1.4	4.6	0.1	0.3	0.2	1.1	49.4	58.7	778.5	2.3
Factor site																	
Kreisbach (All)	494.9^(^*^)^	4.5	333.2	9.1	0.6^(^*^)^	1.0	13.4	1.2	4.7	0.1	0.3	0.3	1.8	36.3	54.6	880.9	0.9
Frauschereck (All)	508.0	7.4	364.1	8.5	0.7	0.9	8.1	0.9	2.6	0.2	0.1	0.1	1.0	44.2	60.2	748.8	1.5

Two one-way ANOVAs were performed to test initial chemical differences between spruce and beech litter and between Kreisbach and Frauschereck (*N* = 2 sites × 2 litter mixtures = 4); only significant results are shown as: ^(^*^)^: *p* < 0.10; *: *p* < 0.05; **: *p* < 0.01; ***: *p* < 0.001

### Mass loss

**Fig. 1:**
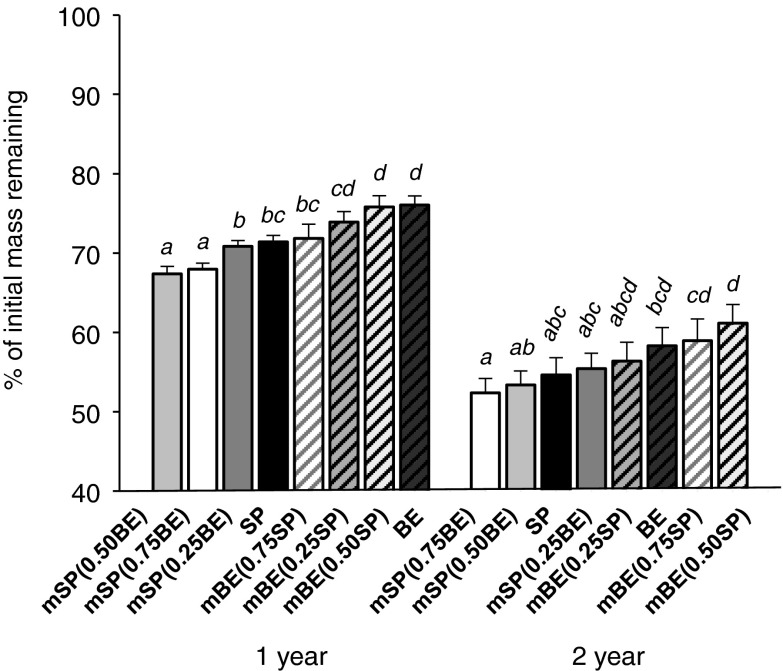
Remaining mass (% of initial values) of exposed litter mixtures in single and mixed species litter bags of spruce and beech. Litterbags were filled with five different mixtures, yielding eight components to be analyzed: single spruce, SP; spruce-beech ratios of 0.75:0.25, mSP(0.25BE), mBE(0.75SP); of 1:1, mSP(0.50BE), mBE(0.50SP); of 0.25:0.75, mSP(0.75BE), mBE(0.25SP); single beech, BE. A three-way (2 × 3 × 8) ANOVA (factors site, incubation stand and litter mixture) was performed for each sampling date after 1 and 2 years (see Table 4). *Plotted bars* represent group means of each litter mixture component (standard errors were calculated for *N* = 2 sites × 3 incubation stands × 4 replications per stand = 24) and different *letters* indicate significant differences between them (Duncan multiple range test, *p* < 0.05)

During the first year of exposure the remaining mass of incubated litter was affected by incubation stand, followed by site and litter mixture (ranked in the order of decreasing *F*-values; Table 4). During the second year of the experiment the incubation stand turned out to be the main controlling factor of mass loss (100 - remaining mass in %), though there was still a small effect of litter species and mixture. Surprisingly, soil chemistry (site) did not affect mass loss after a relatively short time period. However, significant interactions between site and incubation stand and between site and litter mixtures continued, indicating that these factors could not be tested individually. It is striking that these small but significant differences between the 8 individual litter mixture components (litter mixture) did not vary with stand composition (incubation stand), since there was no interaction between these two factors. Overall group means of each litter mixture component (*N* = 24 per sampling date) and results of the attached post-hoc multiple comparisons (Duncan multiple range test) are plotted in Fig. 1. During the first year single beech decayed significantly slower than single spruce litter, however, in all cases the mixed litter decayed faster than the single litter within the same species. After 2 years, significant differences between the remaining mass of single spruce and single beech litter ceased and there was no clear trend visible any longer that mixing speeds up decay of the same species. The neat story about Fig. 1 is the fact that single and mixed spruce litter (needle litter) components occupy the 4 lower ranks (meaning faster decay) and the single and mixed beech litter (leaf litter) components the 4 higher ranks (meaning slower decay) for each sampling date.

**Table 4:** ANOVA table of *F*-values on the effects of site (nutrient-rich soils at Kreisbach versus nutrient-poor soils at Frauscherck), incubation stand (spruce, mixed, beech) and litter mixture on the remaining mass (% of initial values) of litter enclosed in mesh bags. Litterbags were filled with five different mixtures, yielding eight components to be analyzed: single spruce, SP; spruce-beech ratios of 0.75:0.25, mSP(0.25BE), mBE(0.75SP); of 1:1, mSP(0.50BE), mBE(0.50SP); of 0.25:0.75, mSP(0.75BE), mBE(0.25SP); single beech, BE

Effects	df	*F*	*p*	*F*	*p*
		1 year		2 year	
Site (S)	1	15.4	0.0001	0.1	0.7059
Incubation stand (I)	2	33.0	<0.0001	89.3	<0.0001
Litter mixture (L)	7	12.4	0.0000	3.1	0.0047
S × I	2	3.5	0.0343	8.4	0.0004
S × L	7	4.0	0.0005	2.2	0.0388

A three-way (2 × 3 × 8) ANOVA was performed for each sampling date after 1 and 2 years (*N* = 2 sites × 3 incubation stands × 8 litter mixture components × 4 replications per stand = 192). Only significant interactions between the grouping factors are shown, indicating that these factors can not be tested individually but affect the dependent factor jointly

Finally, the average remaining mass after 2 years of decomposition amounted to 67 % (spruce), 54 % (mixed) and 48 % (spruce > mixed > beech), indicating the strong impact of the incubation stand (Table 5). Mean remaining mass was the same for both sites (56 %) due to the declining influence of soil type over time, as discussed above. Performing a one-way ANOVA (factor litter mixture), using the pooled stand means of 4 replications did not show any significant differences between the 8 litter mixture components any more.

**Table 5:** Remaining mass and element contents (% of initial values) and selected compound ratios of litter after 2 years of decomposition for the grouping factors site (nutrient-rich soils at Kreisbach versus nutrient-poor soils at Frauscherck), incubation stand (tree species composition) and litter mixture (litter species composition: single spruce, SP; mixed spruce with 25 % [mSP(0.25BE)], 50 % [mSP(0.50BE)] and 75 % [mSP(0.75BE)] admixture of beech; mixed beech with 25 % [mBE(0.25SP)], 50 % [mBE(0.50SP)] and 75 % [mBE(0.75SP)] admixture of spruce; single beech, BE)

Parameter	Site		Incubation stand			Litter mixture							
	Kreisbach	Frauschereck	Spruce	Mixed	Beech	SP	mSP(0.25BE)	mSP(0.50BE)	mSP(0.75BE)	mBE(0.25SP)	mBE(0.50SP)	mBE(0.75SP)	BE
Mass	56.0	56.4	66.9*c*	53.5*b*	48.2*a*	54.6	55.3	53.3	52.3	56.2	60.9	58.8	58.1
C_org_	49.7	53.4**	61.9*c*	49.5*b*	43.4*a*	51.2	52.0	50.5	49.7	50.4	54.1	52.6	52.0
Lignin	72.4	59.2***	80.1*b*	60.5*a*	56.9*a*	76.1*b*	76.2*b*	71.2*ab*	69.3*ab*	56.0*a*	60.6*ab*	59.2*ab*	57.8*ab*
N_tot_	84.1	108.9**	110.3*b*	92.5*a*	86.7*a*	86.5*abc*	84.7*ab*	76.7*a*	75.1*a*	109.9*bc*	116.8*b*	112.8*bc*	109.3*bc*
P	77.7	73.3	87.8*c*	74.4*b*	64.3*a*	67.3	69.9	72.9	79.4	77.7	78.0	75.2	83.6
S	90.7	93.3	112.0*c*	87.1*b*	76.7*a*	99.7	96.7	97.2	103.3	81.8	87.0	84.9	85.1
Ca	47.6	50.9	56.0	47.9	44.0	36.4*a*	48.5*ab*	37.5*a*	35.2*a*	58.2*ab*	60.8*ab*	53.2*ab*	64.7*b*
Mg	52.3	48.4	57.0*b*	50.3*ab*	43.7*a*	49.0	59.4	55.1	48.0	47.4	50.1	42.6	51.1
K	16.8	18.9	21.6*b*	17.7*a*	14.1*a*	15.1	18.6	18.6	17.8	16.6	18.3	17.9	19.8
Na	132.9	55.8***	125.8*b*	79.0*a*	78.2*a*	66.4	75.7	85.3	58.4	127.5	107.1	106.0	128.2
Al	370.4	239.3**	457.4*b*	209.0*a*	248.0*a*	239.8	265.6	165.9	205.1	355.1	446.6	433.5	327.0
Fe	367.6	433.0	642.8*b*	285.1*a*	272.9*a*	368.0	435.2	360.7	506.3	333.7	423.9	427.0	347.4
Mn	76.7	59.1	80.1	66.3	57.2	33.0*a*	39.6*a*	30.3*a*	27.9*a*	95.0*b*	110.3*b*	112.8*b*	94.0*b*
Lignin/N_tot_	32.3	23.3***	30.6	26.2	26.6	28.2	28.0	30.5	33.3	25.3	25.7	25.9	25.8
C_org_/N_tot_	34.5	30.1	35.2	31.8	30.0	33.8*ab*	34.5*ab*	38.4*ab*	42.2*b*	27.1*a*	27.3*a*	27.5*a*	27.8*a*
C_org_/P	576.5	550.4	581.4	541.2	567.6	652.4*d*	642.1*cd*	608.5*bcd*	549.9*abc*	499.9*a*	537.0*ab*	537.1*ab*	480.2a

Because of insufficient degree of freedom a three-way (2 × 3 × 8) ANOVA could not be performed. Hence, statistics are given for the factors site and incubation stand according to a two-way ANOVA and for the factor litter mixture according to a one-way ANOVA (*N* = 2 sites × 3 incubation stands × 8 litter mixture components × 1 pooled stand mean of four replicated litter bags = 48). Only significant differences between Kreisbach and Frauschereck (factor site) are shown as: *: *p* < 0.05; **: *p* < 0.01; ***: *p* < 0.001. Significant results of a Duncan multiple range test are given for the grouping variables incubation stand and litter mixture (different letters indicate significant differences, *p <* 0.05; *a* represents the lowest mean)

### Nutrient release

Hence, due to the reduced number of replications the same three-way ANOVA performed on remaining mass (Table 4, Fig. 1) had to be split up into a two-way ANOVA (factors site and incubation stand) and a one-way ANOVA (factor litter mixture and comparison of means by a Duncan multiple range test) to test factors controlling the remaining element contents (% of initial value) and selected compound ratios of litter enclosed in mesh bags over a 2-year period (Table 5).

The factor site had little effect (*F*-value: 9.36) on remaining carbon contents but no effect at all on remaining masses (see above), though both showed the same patterns and similar absolute values (Table 5). The soil type (factor site) further controlled the remaining contents of lignin, N_tot_, Na and Al. Stand composition (factor incubation stand) explained the variation in nutrient release (100 - remaining nutrient content in %) for all analyzed elements except Ca and Mn. In cases where nutrient release was affected both by soil type and stand mixture, the latter explained most of the variation according to the *F*-values, except for N_tot_ and Na (Table 5).

Multiple range tests (one-way ANOVA; factor litter mixture) indicated different decreases of lignin (degradation) and nutrient releases for N_tot_, Ca and Mn (expressed in % of initial values) between the 8 litter mixture components. Similar initial C_org_/N_tot_ and C_org_/P ratios (Table 3) for needle and leaf litter declined within 2 years of decomposition, resulting in lower end-values for leaf than for needle litter. However, not a single parameter, listed in Table 5, showed significant difference between single spruce - SP - and mixed spruce litter - mSP(0.25BE), mSP(0.50BE), mSP(0.75BE) - or between single beech - BE - and mixed beech litter - mBE(0.25SP), mBE(0.50SP), mBE(0.75SP) - with the exception for the C_org_/P ratio in needle litter. Ranking the C_org_/P ratios within incubated mesh bags after 2 years from high to low corresponds to ranking the admixture of beech from 0 to 100 %, indicating nutrient transfer processes from one species to the other within mixed bags. For all other elements or element ratios, direct effects via nutrient transfer among litter of the different species seem unlikely, since these differences (grouping variable litter mixture) were only measured between leaves and needles in general.

So far, we expressed nutrient release (and remaining nutrient content, respectively) in % of initial values, because this unit seems more appropriate for reporting relative changes caused by litter mixing. However, especially for elements with different initial litter contents between spruce and beech (see Table 3), absolute numbers on nutrient release in mg g^−1^ incubated litter may yield different statistical results. Running a multiple range test attached to the one-way ANOVA of Table 5 with absolute instead of relative units yielded only one homogenous group for Ca (4.53–6.21) but two homogenous groups for Mg (needle litter, *a*: 0.24–0.32; leaf litter, *b*: 0.69–0.81) and K (needle litter, *a*: 2.21–2.32; leaf litter, *b*: 3.73–3.84; all data in element release in mg g^−1^ incubated litter). For all other elements the number of homogenous groups (as result of a multiple range test) did not change. All data given in this paper as remaining nutrient content in % of initial value can be easily converted to nutrient release in mg g^−1^ incubated litter by multiplying nutrient release in % (100 - remaining nutrient content in %) with initial litter chemistry in mg g^−1^ (given in Table 3).

As reported elsewhere (e.g., Prescott et al. 1993; Albers et al. 2004) nutrient immobilization during the early phases of decomposition may be followed by release of the same nutrient during later phases. Net remaining nutrient contents above 100 % after 2 years of decomposition for N_tot_ and Mn (leaf litter), S (needle litter) and Al and Fe (both litter types) clearly indicate immobilization processes (Table 5), corresponding to negative absolute releases in mg g^−1^ litter. The observed net immobilization of Al and Fe was in accordance with Schlesinger (1997), reporting that plant litter appears to absorb Al and Fe, perhaps in compounds that are precursors to the fulvic acids.

### Prediction of mixed litter decomposition

**Fig. 2:**
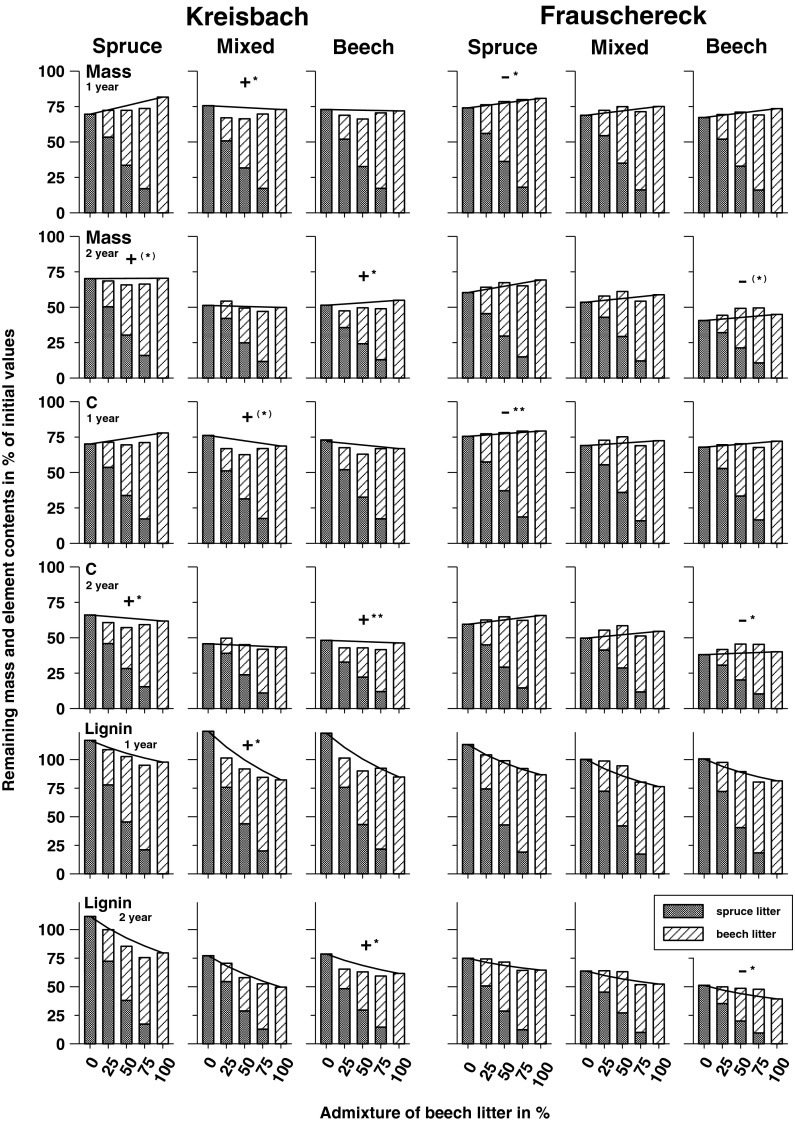
Remaining mass and contents of C_org_ and lignin (% of initial values) of exposed litter mixtures in single and mixed species litter bags of spruce and beech after 1 and 2 years, grouped by site (Kreisbach, Frauscherck) and incubation stand (spruce, mixed, beech). Litterbags were filled with five different mixtures, plotted on the x-axis in the order of increasing admixture (% of initial mass) of beech. Each column represents just one pooled stand mean of four replicated litter bags. Predicted values, based upon linear interpolation from single species mixtures of spruce and beech, are potted as *lines*. Significant differences (paired *t*-test; *N* = 3) between predicted and measured total (mixed spruce + mixed beech within a bag) remaining masses and element contents in mixed litter bags of all three combinations (25, 50 and 75 % admixture of beech) are indicated + (if positive) or – (if negative); level of significance is shown as: ^(^*^)^: *p* < 0.10; *: *p* < 0.05; **: *p* < 0.01; ***: *p* < 0.001

**Fig. 3:**
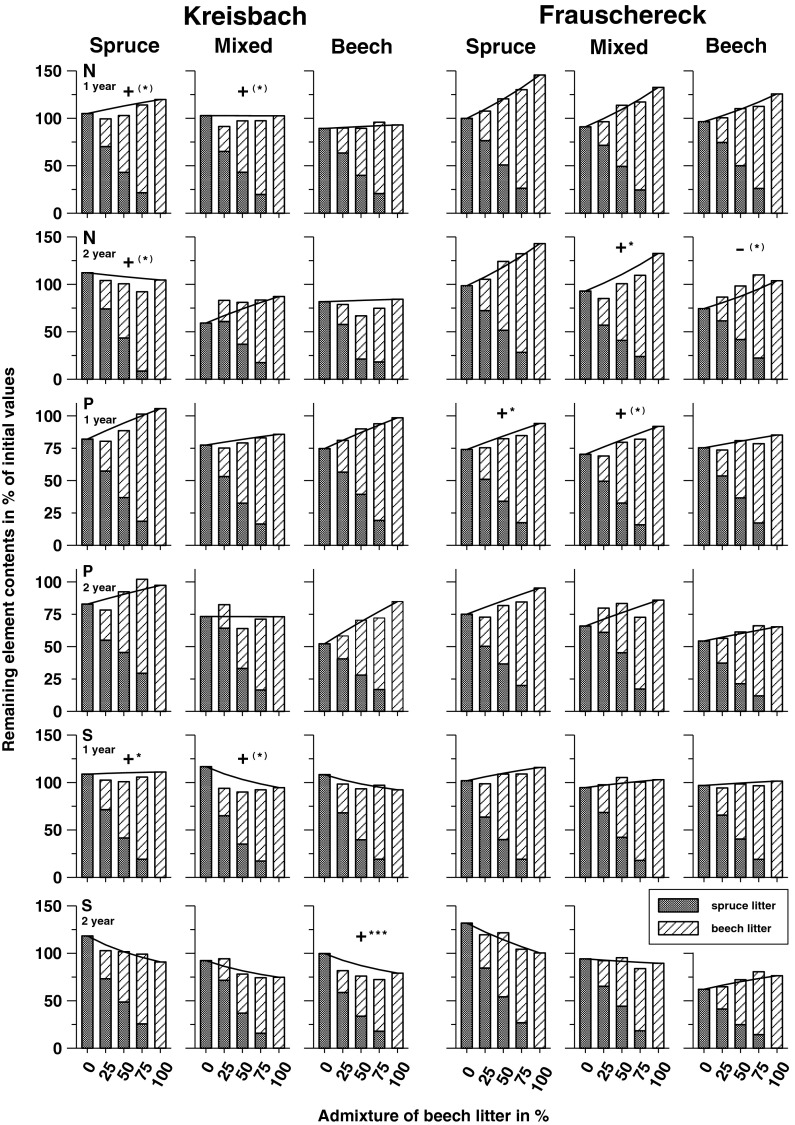
Remaining contents of N_tot_, P and S (% of initial values) of exposed litter mixtures in single and mixed species litter bags of spruce and beech after 1 and 2 years, grouped by site (Kreisbach, Frauscherck) and incubation stand (spruce, mixed, beech). Litterbags were filled with five different mixtures, plotted on the x-axis in the order of increasing admixture (% of initial mass) of beech. Each column represents just one pooled stand mean of four replicated litter bags. Predicted values, based upon linear interpolation from single species mixtures of spruce and beech, are potted as *lines*. Significant differences (paired *t*-test; *N* = 3) between predicted and measured total (mixed spruce + mixed beech within a bag) remaining element contents in mixed litter bags of all three combinations (25, 50 and 75 % admixture of beech) are indicated + (if positive) or – (if negative); level of significance is shown as: ^(^*^)^: *p* < 0.10; *: *p* < 0.05; **: *p* < 0.01; ***: *p* < 0.001

**Fig. 4:**
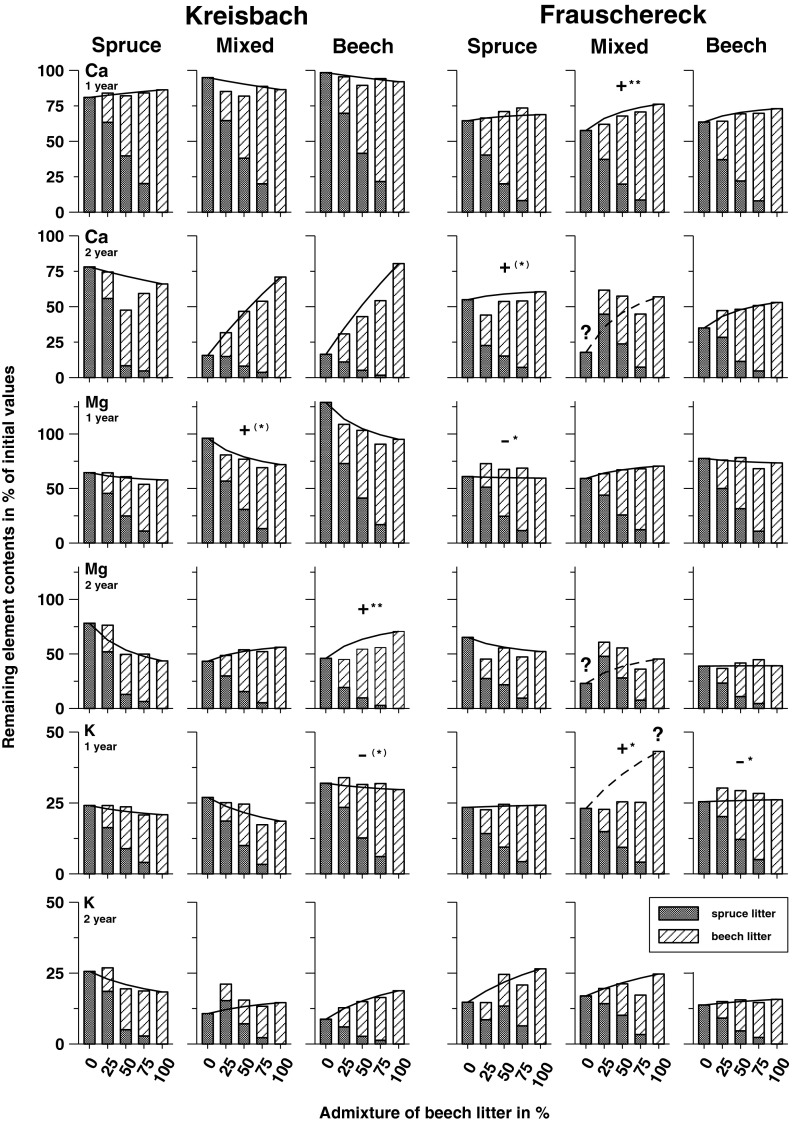
Remaining contents of Ca, Mg and K (% of initial values) of exposed litter mixtures in single and mixed species litter bags of spruce and beech after 1 and 2 years, grouped by site (Kreisbach, Frauscherck) and incubation stand (spruce, mixed, beech). Litterbags were filled with five different mixtures, plotted on the x-axis in the order of increasing admixture (% of initial mass) of beech. Each column represents just one pooled stand mean of four replicated litter bags. Predicted values, based upon linear interpolation from single species mixtures of spruce and beech, are potted as *lines*. Significant differences (paired *t*-test; *N* = 3) between predicted and measured total (mixed spruce + mixed beech within a bag) remaining element contents in mixed litter bags of all three combinations (25, 50 and 75 % admixture of beech) are indicated + (if positive) or – (if negative); level of significance is shown as: ^(^*^)^: *p* < 0.10; *: *p* < 0.05; **: *p* < 0.01; ***: *p* < 0.001. *Question marks* indicate that the corresponding single species contents look like outliers, though no mistakes were found. Hence, calculated predicted values (*dotted lines*) must be interpreted with caution

Direct effects via nutrient transfer of the studied elements among litter of the different species were excluded (compare Table 5). However, indirect effects (e.g., changing decomposer abundance and activity) were partly visible for total (mixed spruce + mixed beech litter) remaining masses and element contents in the mixed litter bags, deviating from predicted values based upon single species mixtures of spruce and beech (this means, mass loss or nutrient release of both components within mixed bags may be driven jointly in either way). Out of 108 cases plotted in Figs. 2, 3 and 4 (grouped by remaining mass, C, lignin and macro nutrients; year; site and incubation plot), litter mixtures displayed additive characteristics in 78 cases and non-additive characteristics in 30 cases. Out of the 30 (non-additive) cases, 21 cases were characterized by faster and 9 cases by slower decomposition rates than predicted. It is striking that out of these latter 9 cases only one case was recorded for K at Kreisbach (1 year, beech stand) while the majority of cases with retarded decomposition in the mixed bags occurred at the nutrient-poor soils at Frauschereck. We suggest that the decomposer diversity and abundance is not high enough (compare significantly lower C_mic_/C_org_ ratios in Table 2) to react immediately to freshly cross-transplanted litter.

Figures 2, 3 and 4 summarize the whole data set of the study, broken down on the lowest level cases: pooled stand means of 4 replicated sets of the five litter mixtures, which were connected with each other by two strings (see Materials and Methods section). Due to a shortage of replications for specific statistical tests, frequency analyses, e.g., via simple counting of hastened vs. slowed decomposition cases, helps to draw justified conclusions. Though each column is based upon just one chemical analysis it is easy to detect outliers visually, since the litter bag results are plotted on the x-axis in the order of increasing admixture of beech (% of initial mass) for each case. Out of 864 plotted bars only 3 look like possible outliers or extreme values (question-marked but kept in the graphs, since no mistakes were found). Predicted values of remaining masses and nutrient contents in % of initial values are based upon linear interpolation from single species mixtures of spruce and beech and were plotted as line graph for each case. Note that litterbags were plotted in the order of increasing additions of leaf litter masses; consequently, deviations from a straight line are an indication that initial foliar chemistry was different between spruce and beech (compare Table 3).

We tried to summarize non-additive effects via grouping all 108 cases by site and year in Table 6, since we know that the effect of litter mixture on mass loss (non-pooled samples) did not vary with incubation stand (see above). Significant paired *t*-tests indicated without exception higher decomposition rates than predicted in the mixed litter bags at the nutrient-rich site Kreisbach, but slower rates than predicted for the nutrient-poor site Frauschereck. Performing the same tests on the whole data set for each year indicated that mass loss and release of C_org_, N_tot_, P, S and Ca was hastened within the first year of decomposition but ceased thereafter. It is important to point out that non-additive effects of mixed litter were measured, though no differences between the single species were visible for most parameters.

**Table 6:** Significant mean differences (paired *t*-test) between predicted (linear interpolation from single species mixtures of spruce, SP, and beech, BE) and measured total (mixed spruce + mixed beech within a bag) remaining masses and element contents (% of initial values) in mixed litter bags of all 3 combinations (25, 50 and 75 % admixture of beech), corresponding single species data for SP and BE and ranked differences of specific mixtures

Site	Parameter	Predicted—measured	SP	BE	Ranked differences
Year		All mixed bags	Single species bags		Specific mixtures
Kreisbach					
1 year	Mass	4.4**	72.7	75.6 ns	75, 25, 50 ns
	C_org_	4.7*	72.8	71.2 ns	75, 25, 50 ns
	Lignin	6.0**	121.5	88.2**	75, 25, 50 ns
	N_tot_	4.8**	99.1	105.2 ns	75, 50, 25 ns
	S	7.1**	111.2	99.3 ns	75, 25, 50 ns
	Ca	2.5^(^*^)^	91.5	88.3 ns	75, 25, 50 ns
	Mg	3.1*	96.5	75.0 ns	50, 25, 75 ns
2 year	Mass	2.7*	57.6	58.5 ns	25, 50, 75 ns
	C_org_	2.9*	53.3	50.5 ns	25, 50, 75 ns
	Lignin	4.5**	89.1	63.6 ns	25, 50, 75 ns
	Ca	6.9*	36.8	72.5 ns	25, 75, 50 ns
Frauschereck					
1 year	P	3.6**	73.2	90.5**	50 < 75, 25*
2 year	Mass	−2.4^(^*^)^	51.5	57.7 ns	50, 25, 75 ns
	C_org_	−2.9*	49.1	53.4 ns	50, 25, 75 ns
	Lignin	−2.8*	63.2	52.0 ns	50, 25, 75 ns
All					
1 year	Mass	2.0*	71.4	76.0^(^*^)^	25, 50, 75 ns
	C_org_	2.0*	71.8	72.9 ns	25, 75, 50 ns
	N_tot_	3.0**	97.5	119.9*	50, 75, 25 ns
	P	2.7**	75.7	93.6**	50, 75, 25 ns
	S	4.4**	104.5	103.0 ns	75, 50, 25 ns
	Ca	1.7^(^*^)^	76.7	80.5 ns	75, 25, 50 ns

Differences between predicted and measured values for specific mixtures were compared by a Duncan multiple range test (*p* < 0.05) and ranked in increasing order, if not significant (*N* per site and year = 3 incubation stands × 3 mixed bags × 1 pooled stand mean of four replicated litter bags = 9). The corresponding single species data are given for spruce (SP) and beech (BE) and means are compared via a one-way ANOVA (*N* per site and year = 3 incubation stands × 2 single species bags × 1 pooled stand mean of four replicated litter bags = 6)

Differences between predicted and measured values for specific mixtures (25, 50 and 75 % admixture of beech) were compared by a Duncan multiple range test to see which mixture had the biggest non-additive effect. With the exception of P (1 year, Frauschereck) none of the multiple comparisons yielded significant results. There was a trend that relatively low admixtures of beech (25 %) or spruce (corresponding to 75 % admixture of beech) displayed the highest non-additional characteristics at Frauschereck (see column “ranked differences” in Table 6).

## Discussion

### Question 1: Does beech litter decompose faster than spruce litter?

i) Mass loss of single beech litter was not higher than mass loss of single spruce litter. During the first year decay of single beech litter was significantly lower than of single spruce, but differences declined over time.

ii) Net nutrient release in mg g^−1^ incubated litter (after 2 years) of N_tot_, and Mn was higher in needle than in leaf litter due to high immobilization (retention) rates of beech.

iii) However, decrease of lignin and release of Mg and K (mg g^−1^) were higher for leaf than needle litter.

Again, the common (implicit) expectation that the broadleaf components decay faster was not fulfilled in accordance with our overall approach (Berger and Berger 2012). Slower decay of beech versus spruce litter is in accordance with Vesterdal (1999; at one of 3 sites only), Albers et al. (2004) and Sariyildiz et al. (2005; comparison between *Fagus orientalis* and *Picea orientalis*). This finding agrees with Prescott et al. (2004) and Prescott (2010), stating that many broadleaf species do not decay faster than needles, and if they do this difference is only evident for the first 1–3 years, after which time a similar or even greater proportion of the original mass of broadleaf litter may remain compared with needle litter. In accordance with Dungait et al. (2012) lignin is not as recalcitrant as thought. It has to be pointed out that the structure of lignin varies between tree species (a good overview of initial chemical patterns and their changes during lignin degradation is given for beech leaves and spruce needles by Klotzbücher et al. 2011). Obviously, the purported faster decomposition of beech leaf litter, as often reported in German textbooks (e.g., Ellenberg et al. 1986; Rehfuess 1990), is not a safe generalization to make and, maybe, was deduced from the fact that fresh foliage of beech is of better quality than of spruce. E.g., comparisons between beech and spruce foliage at the 6 mixed beech-spruce stands within the overall project by Berger et al. (2009a) indicated significantly higher nutrient concentrations of beech foliage for all elements (except for Mn, and P at nutrient-rich sites only). Retranslocation of nutrients prior to senescence (Kristensen et al. 2004; Berger et al. 2009b; Carnol and Bazgir 2013) is greater in beech than in spruce foliage, which may have caused the negligible differences between initial spruce and beech litter in this study. We did not expect fresh spruce and beech litter to be of similar quality; under this precondition the similar decay rates measured are not surprising, since most of the other factors influencing decomposition processes (macro- and micro-climate, soil organisms and soil nutrient status) were the same on transplanted litter bags.

### Question 2: Does litter decompose faster in beech or mixed beech-spruce forests than in spruce forests?

i) Decay and C_org_ release were primarily affected by tree species composition of the incubation stand and were faster in (mixed) beech forests stands than in spruce forests. Litter decay indicated non-additive patterns at the nutrient-rich site Kreisbach, since similar remaining masses under pure beech (50 %) and mixed beech-spruce (50 %) were significantly lower than under pure spruce stands (68 %; beech = mixed < spruce), however, linear (additive) reactions as expected from the pure stands at the nutrient-poor site Frauschereck: beech (46 %) < mixed (57 %) < spruce (66 %).

ii) Remaining element contents in % of initial values after 2 years increased for all elements (except Ca) significantly from beech over mixed to spruce stands and showed non-additive patterns at Kreisbach (beech = mixed < spruce) but additive patterns (beech < mixed < spruce) at Frauschereck for C_org_, lignin, S, K and Fe.

Although beech litter itself did not decay faster than spruce litter within 2 years, favorable environmental conditions in (mixed) beech stands increased litter decay. This is in accordance with the overall approach. However, very similar remaining masses were recorded within 3 years (beech: 47 %; mixed: 48 %; spruce: 67 %; means of 6 sites; Berger and Berger 2012) as within the 2 monitoring years of this study (beech: 48 %; mixed: 54 %; spruce: 67 %; means of 2 sites). Obviously, decay rates can vary strongly depending on climatic conditions. It is of practical relevance to know that the formation of thick organic layers in spruce monocultures, suggested to hamper productivity, can be avoided by admixture of beech and does not necessarily require complete stand conversion at nutrient-rich sites.

### Question 3: Does mixing of beech and spruce litter hasten decomposition?

i) In general, the impact by litter species and their mixtures on decay rates was very small and was mainly driven by stand mixture.

ii) During the first year mixed litter decayed faster than the single litter within the same species, however, this visible trend that mixing speeds up decay ceased after 2 years.

iii) Direct effects via nutrient transfer among litter of the different species seemed unlikely, since all elements displayed the same release between single spruce and mixed spruce litter and between single beech and mixed beech litter, respectively. Nevertheless, in some cases indirect effects (e.g., changing decomposer abundance and activity) may explain the fact that non-additive pattern were visible for the totals (mixed spruce + mixed beech litter) within the mixed bags, driving decomposition of both components jointly in either way. E.g., mass loss and release of C_org_, N_tot_, P, S and Ca was hastened within the first year of decomposition but ceased thereafter. At the nutrient-poor site Frauschereck retarded decomposition in the mixed litter bags was recorded in a few cases as well.

It is hypothesized that enhanced decay rate and nutrient release in mixtures of litter, as shown by a number of authors (Gartner and Cardon 2004 and references therein), is caused by translocation of nutrients between litters of different quality (direct effects), resulting in a more rapid and efficient utilization of litter substrate by decomposers. However, in accordance with Berger and Berger (2012), net transfers of nutrients between the two litter species in the mixed bags were minimal, since initial beech and spruce litter did not reveal different litter quality. Clear indirect effects on decomposition caused by stand mixture can not be mimicked by litter mixtures within mesh bags.

## Conclusions

This study verified the conclusion of Berger and Berger (2012) that “greater accumulation of litter in spruce compared to beech stands is not a consequence of the inherent recalcitrance of needles”. Faster decomposition of beech litter is not a safe generalization to make, and is obviously not the cause of the differences in soils beneath the two species. In accordance with Albers et al. (2004) we conclude that adverse environmental conditions in spruce stands retard decomposition. Mixed beech-spruce stands appear to be effective in counteracting these adverse conditions, preventing the accumulation of thick organic layers observed in spruce monocultures.
